# Clozapine treatment during the COVID-19 pandemic

**DOI:** 10.1192/j.eurpsy.2021.1777

**Published:** 2021-08-13

**Authors:** E. Dąbrowska, B. Galińska-Skok, D. Zalewski, A. Nobis

**Affiliations:** Department Of Psychiatry, Medical University of Bialystok, Choroszcz, Poland

**Keywords:** COVID-19, treatment-resistent schizophrenia, clozapine, coronavirus

## Abstract

**Introduction:**

Clozapine is an effective antipsychotic used in treatment-resistant schizophrenia. One of the serious complications of clozapine therapy is agranulocytosis, therefore regular monitoring of the level of white blood cells (WBC) in plasma is necessary. During acute inflammatory infections, including the COVID-19 infection, levels of clozapine may increase, by the CYP 450 system, leading to adverse effects such as sedation, hypersalivation and consequently to aspiration pneumonia.

**Objectives:**

The aim of the study is to assess the validity of continuing clozapine treatment during the COVID-19 pandemic.

**Methods:**

Brief literature review, based on research of scientific articles published in PubMed, using as keywords the terms “clozapine” and “COVID-19”.

**Results:**

Research studies indicate that SARS-COV2 infection in patients treated with clozapine does not significantly reduce the level of neutrophils, despite commonly observed leucopenia with lymphopenia. Due to pandemic, existing WBC scheme has been modified with a trend towards less frequent measurements. To minimize the side effects of clozapine during infection, it is recommended to reduce the dose of clozapine by half and continue lower dose until 3 days after fever has subsided. Additionally, there have been reports of psychosis recurrence after discontinuation of clozapine causing difficulties in treatment COVID-19 as also comorbid mental disease.

**Conclusions:**

Treatment of clozapine is associated with higher risk of COVID-19 complications and at the same time COVID-19 infection may increase clozapine toxicity. Therefore, the risks of COVID-19 infection present a challenge for safe clozapine use. Further research will be needed to assess these dependencies and strategies.

**Disclosure:**

No significant relationships.

